# Prognostic Significance of Delays in Initiation of Adjuvant Trastuzumab-Based Therapy in Patients with HER2-Positive Breast Cancer

**DOI:** 10.3390/biomedicines13061305

**Published:** 2025-05-26

**Authors:** Gavin P. Dowling, Aisling Hegarty, Gordon R. Daly, Sandra Hembrecht, Cian M. Hehir, Gavin G. Calpin, Richard Hogan, David O’Reilly, Eithne Downey, Sinead Toomey, Liam Grogan, Oscar Breathnach, Michael Allen, Patrick G. Morris, Colm Power, Leonie S. Young, Arnold D. K. Hill, Bryan T. Hennessy

**Affiliations:** 1 Department of Surgery, Royal College of Surgeons in Ireland, RCSI University of Medicine and Health Sciences, D02 YN77 Dublin, Ireland; aislinghegarty@rcsi.com (A.H.); gordondaly@rcsi.com (G.R.D.); sandrahembrecht@rcsi.com (S.H.); cianhehir23@rcsi.com (C.M.H.); gavincalpin22@rcsi.com (G.G.C.); richardhogan@rcsi.com (R.H.); michaelallen@rcsi.ie (M.A.); cpower@rcsi.com (C.P.); lyoung@rcsi.com (L.S.Y.); adkhill@rcsi.ie (A.D.K.H.); 2 Medical Oncology Laboratory, Department of Molecular Medicine, RCSI University of Medicine and Health Sciences, D02 YN77 Dublin, Ireland; sineadtoomey@rcsi.com (S.T.); bryanhennessy74@gmail.com (B.T.H.); 3 Beaumont RCSI Cancer Centre, Beaumont Hospital, D09 FT51 Dublin, Ireland; davidoreilly22@rcsi.com (D.O.); eithnedowney@beaumont.ie (E.D.); liamgrogan@rcsi.ie (L.G.); oscarbreathnach@rcsi.ie (O.B.); patrickmorris@rcsi.com (P.G.M.)

**Keywords:** breast cancer, trastuzumab, adjuvant therapy, targeted therapy, breast surgery

## Abstract

**Purpose:** Adjuvant trastuzumab therapy has improved outcomes in HER2-positive breast cancer, but the impact of the timing of its initiation remains unclear. This study evaluates the effect of time to adjuvant trastuzumab-based therapy (TTAT) after surgery on survival in HER2-positive breast cancer. **Methods:** In this retrospective study, HER2-positive breast cancer patients treated with surgery followed by adjuvant trastuzumab without prior neoadjuvant therapy were analyzed. Patients were grouped by TTAT ≤ 42 days or >42 days post-surgery. Key endpoints included overall survival (OS), disease-free survival (DFS), and distant metastasis-free survival (DMFS), evaluated through Kaplan–Meier and Cox regression analyses. **Results:** Patients with TTAT greater than 42 days had significantly worse OS, DFS, and DMFS (*p* = 0.036, *p* = 0.045, and *p* = 0.048, respectively, log-rank test) than those initiating trastuzumab within 42 days. On multivariate analysis, delays beyond 42 days were associated with a significantly increased risk of recurrence and mortality, showing reduced DFS (HR 2.52; *p* = 0.027) and OS (HR 4.48; *p* = 0.004). These findings indicate that even moderate delays in trastuzumab initiation can adversely affect survival. **Conclusions:** Delaying trastuzumab initiation beyond 42 days post-surgery negatively impacts survival in HER2-positive breast cancer, emphasizing the need for timely treatment. These results support clinical guidelines advocating prompt initiation of adjuvant therapy to improve long-term outcomes for HER2-positive patients.

## 1. Introduction

Human epidermal growth factor receptor 2 (HER2) is overexpressed and/or amplified in 15–20% of all breast cancers. Prior to the development of HER2-directed therapies, this subtype was associated with a poor prognosis [1]. The addition of trastuzumab, the first humanized anti-HER2 monoclonal antibody, to adjuvant treatment regimens for patients with HER2-positive breast cancer transformed the treatment landscape. Numerous clinical trials demonstrated the efficacy of trastuzumab, combined with various chemotherapy regimens, in this population. This benefit in efficacy was seen regardless of whether trastuzumab was administered simultaneously with chemotherapy, after an initial cycle, or following its completion [2,3,4,5].

Extensive research has been undertaken to establish the optimal duration of adjuvant trastuzumab; however, less is known about the prognostic significance of timely initiation of trastuzumab therapy after surgery. The clinical impact of delays in the commencement of adjuvant therapies in breast cancer of all subtypes has been investigated in some retrospective studies. Several of these found that extreme delays in initiation of adjuvant therapy negatively impacted survival [6,7,8], while less drastic delays failed to demonstrate this impact [9,10,11]. One retrospective study found that the HER2-positive subgroup had significantly worse overall survival (OS) when adjuvant systemic therapy was initiated ≥61 days after surgery [12]. Another study investigated the effect of time from breast cancer diagnosis to adjuvant trastuzumab in HER2-positive patients, demonstrating superior survival outcomes when trastuzumab is initiated within 6 months of diagnosis [13]. To our knowledge, the most recent of these studies included data up to 2012, with a median follow-up of 3.4 years.

Despite the heterogeneity in the literature, these initial results highlight the possible negative effect of delaying initiation of trastuzumab in the adjuvant setting. Additionally, adjuvant treatment regimens have evolved significantly over the last decade, underscoring the need for investigating this effect in the contemporary setting, with meaningful follow-up. Therefore, this study aimed to explore the impact of timely initiation of adjuvant trastuzumab in a cohort of HER2-positive patients who had not received neoadjuvant therapy.

## 2. Methods

### 2.1. Study Population

A single-center, retrospective analysis was conducted on a prospectively maintained institutional database in an Irish tertiary referral center. Women with stage I to III invasive breast cancer which was HER2-positive in accordance with international guidelines (ASCO/CAP guidelines) at the time of diagnosis and underwent surgery and subsequently received adjuvant trastuzumab-containing therapy were included. Patients who underwent neoadjuvant treatment prior to surgery were excluded from this study. To be eligible for inclusion, accurate dates for diagnosis, surgery, and initiation of adjuvant trastuzumab had to be available.

Data were extracted on patient demographic, tumor, and pathological characteristics. Detailed data on adjuvant treatment regimens and surgery types were also recorded. Local ethical approval was obtained for this study (CA2023/052) from the Beaumont Hospital Ethics Committee, and the need for informed consent was deemed unnecessary. This research was performed in accordance with the Declaration of Helsinki [14].

### 2.2. End Points

Overall survival (OS) was defined as the interval between the initial diagnosis of breast cancer to the date of last follow-up or the date of death from any cause. Disease-free survival (DFS) was calculated from the initial diagnosis to the date of first documented local or distant recurrence or last follow-up. Distant metastasis-free survival (DMFS) was measured from the date of initial diagnosis to the date of first documented distant recurrence or last follow-up. Time to adjuvant trastuzumab-based therapy (TTAT) was defined as the interval between initial surgery for HER2-positive invasive breast cancer and the date of initiation of adjuvant trastuzumab. A cut-off of 42 days (6 weeks) was chosen based on the European Society for Medical Oncology (ESMO) guidelines, which advise commencing adjuvant therapy for breast cancer within 4–6 weeks [15].

### 2.3. Statistical Analysis

Clinicopathological, tumor, and treatment characteristics were analyzed using descriptive statistics; Fisher’s exact (¶), Chi-squared (χ^2^), and one-way analysis of variance (ANOVA, •) tests were used as appropriate. All tests of significance were 2-tailed, with *p* < 0.050 indicating statistical significance. Kaplan–Meier and Log-rank (Mantel–Cox) analyses were performed to determine differences in survival between the patients with TTAT ≤42 versus >42 days. Multivariable Cox proportional hazards regression models were used to estimate the effect of delayed trastuzumab initiation on OS and DFS expressed as hazard ratios (HR) with 95% CIs. The models were adjusted for key confounding variables, including age at diagnosis, tumor size, lymph node status, hormone receptor status, type of surgery, chemotherapy regimen, use of adjuvant hormonal therapy, and receipt of radiotherapy. Variables with *p* < 0.050 in univariable analysis were included in the multivariable analysis. Data were analyzed using Statistical Package for Social Sciences^TM^ (SPSS^TM^) Version 26 (International Business Machines Corporation, Armonk, New York, NY, USA).

## 3. Results

**Figure 1:**
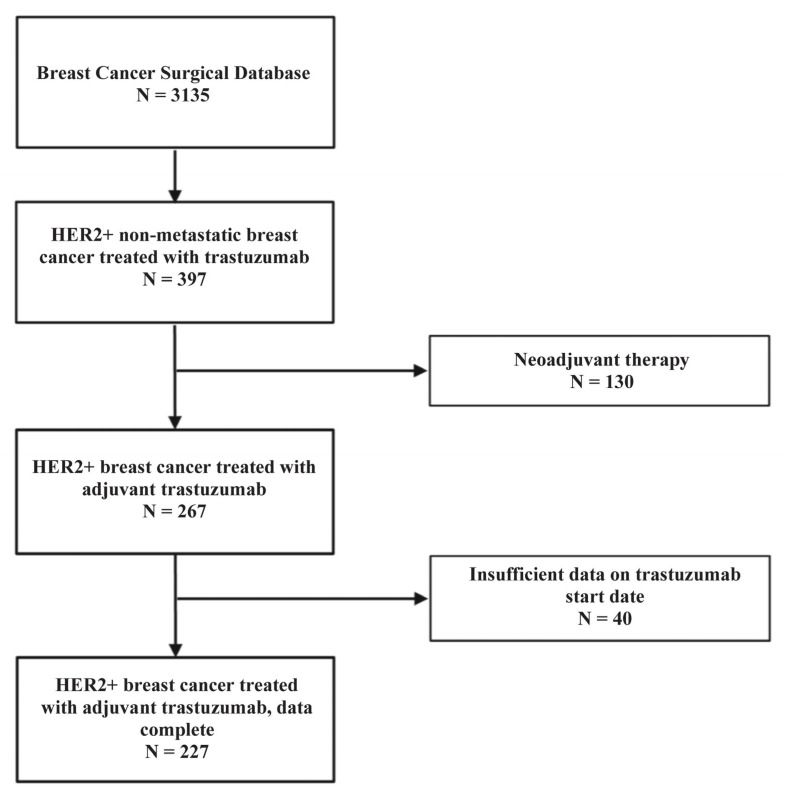
Flowchart outlining the selection of patients from the Breast Cancer Surgical Database for inclusion in the study of HER2-positive non-metastatic breast cancer treated with adjuvant trastuzumab. From an initial cohort of 3135 patients in the database, 397 patients with HER2-positive non-metastatic breast cancer who received trastuzumab therapy were identified. Of these, 130 patients who received neoadjuvant trastuzumab were excluded, resulting in 267 patients who received adjuvant trastuzumab. An additional 40 patients were excluded due to insufficient data regarding the start date of trastuzumab therapy. The final study population included 227 patients with HER2-positive non-metastatic breast cancer treated with adjuvant trastuzumab and complete treatment data.

Out of 3135 patients on the database, 227 patients met the predefined inclusion criteria and formed the overall population of this study. The study flow chart is shown in Figure 1. The patients’ main characteristics are detailed in Table 1. The median time from initial breast cancer diagnosis to trastuzumab initiation was 49 days. The study population was divided into two cohorts based on their TTAT; 80 patients (35.24%) were in the ≤42 days cohort, and 147 (64.76%) were in the >42 days cohort. These cohorts did not have any statistically significant differences in terms of age (*p* = 0.9, •), tumor size (*p* = 0.96, •), T stage (*p* = 0.6, χ^2^), N stage (*p* = 0.12, χ^2^), hormone receptor status (0.27, χ^2^) or tumor grade (*p* = 0.7, χ^2^). There were also no significant differences in terms of the surgery type (*p* = 0.16, χ^2^) or adjuvant regimen (0 = 0.33, χ^2^) received between the groups.

At a median follow-up of 5.2 years, in the overall study population (n = 227), 33 (14.5%) recurrences were observed, and 26 (11.5%) deaths occurred. On univariate Cox regression analysis, patients with N3 stage (HR 4.09; 95% CI: 1.48–11.31, *p* = 0.007), T3 stage (HR 11.05; 95% CI: 1.3–94.09, *p* = 0.028) and negative hormone receptor status (HR 2.69; 95% CI: 1.25–5.8, *p* = 0.012) had a significantly higher risk of death. The risk of recurrence was also significantly greater in patients with N3 stage (HR 3.01; 95% CI: 1.2–7.58, *p* = 0.019), negative hormone receptor status (HR 2.4; 95% CI: 1.21–4.77, *p* = 0.012) and those who received TCH-like adjuvant regimens (HR 3.95; 95% CI: 1.01–7.21, *p* = 0.047).

On multivariate Cox regression analysis, patients with N3 stage (HR 3.32; 95% CI: 1.15–9.95, *p* = 0.026), T3 stage (HR 12.09; 95% CI: 1.25–117.09, *p* = 0.031) and negative hormone receptor status (HR 2.34; 95% CI: 1.07–5.14, *p* = 0.034) had a significantly higher risk of death. However, negative hormone receptor status (HR 2.12; 95% CI 1.05–4.29, *p* = 0.036) was the only variable significantly associated with recurrence on multivariate regression analysis.

**Figure 2:**
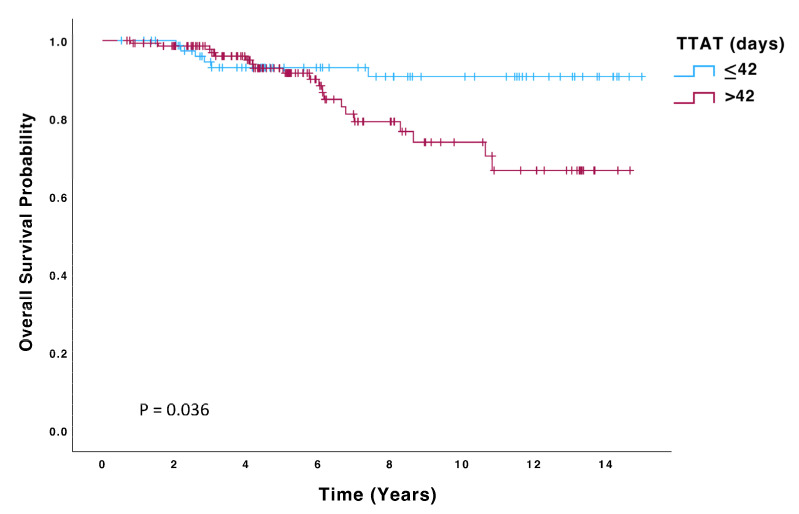
Overall survival (OS) of patients with TTAT ≤ 42 days (n = 80) compared to those with TTAT > 42 days (n = 147).

**Figure 3:**
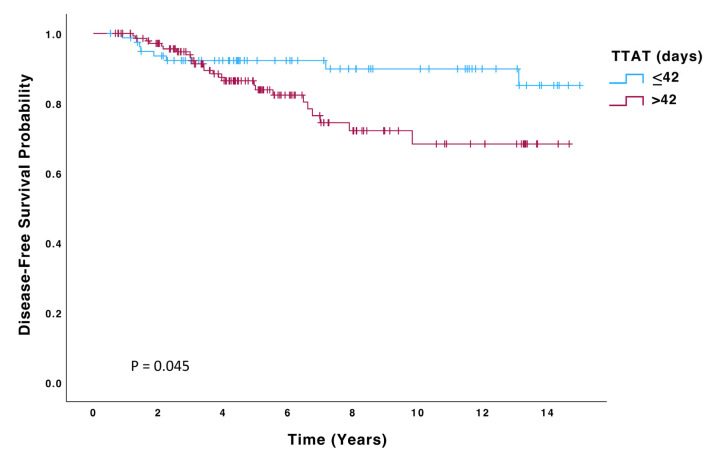
Disease-free survival (DFS) of patients with TTAT ≤ 42 days (n = 80) compared to those with TTAT > 42 days (n = 147).

**Figure 4:**
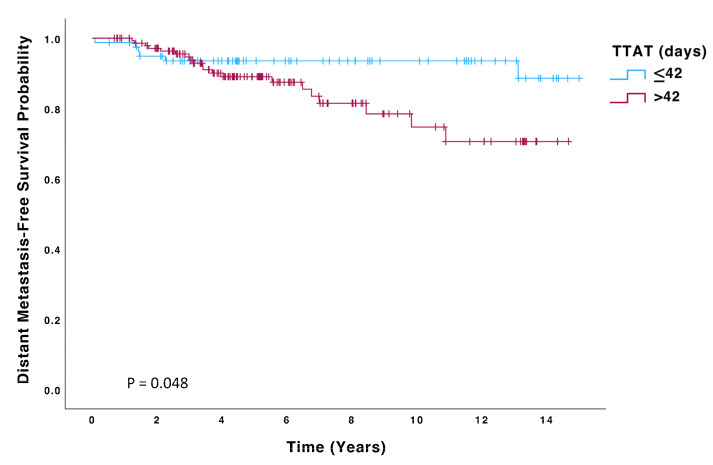
Distant Metastasis-Free Survival (DMFS) of patients with TTAT ≤ 42 days (n = 80) compared to those with TTAT > 42 days (n = 147).

Patients in the > 42-day group had a significantly higher risk of recurrence (HR 2.23; 95% CI: 0.99–4.99, *p* = 0.050) and death (HR 2.59; 95% CI: 1.03–6.51, *p* = 0.043) than those in the ≤42 days group. Kaplan–Meier curves for OS, DFS, and DMFS are displayed in Figure 2, Figure 3 and Figure 4, and significant differences were observed in all these analyses on the log-rank test (*p* = 0.036, *p* = 0.045, *p* = 0.048, respectively). On multivariate Cox regression analysis, significant differences were observed in both OS (HR 4.48; 95% CI: 1.6–12.6, *p* = 0.004) and DFS (HR 2.52; 95% CI: 1.11–5.74, *p* = 0.027).

## 4. Discussion

The benefit of anti-HER2 therapy in early-stage HER2+ breast cancer is significant, irrespective of patient and tumor characteristics, including hormone receptor status [16,17]. Neoadjuvant treatment has become the preferred approach for most of these patients [18,19]. However, primary surgery followed by adjuvant chemotherapy in combination with trastuzumab remains the standard of care for patients with early-stage HER2+ breast cancer, which is <2 cm and clinically node-negative. This is based on results from the APT trial, in which these patients with low tumor burden were treated with 12 weeks of adjuvant paclitaxel and trastuzumab, followed by trastuzumab to complete 12 months of therapy. Impressive 10-year overall and recurrence-free survival were observed in this cohort, 94.3% (95% CI 91.8–96.8) and 96.3% (95% CI 94.3–98.3), respectively [20,21]. While the benefit of adjuvant chemotherapy with trastuzumab is substantial, there is conflicting evidence on the prognostic significance of timely initiation of adjuvant therapy after surgery.

There is pre-clinical evidence that surgical excision of the primary tumor may disseminate tumor cells and produce circulating growth-stimulating factors, potentially leading to an increased risk of metastatic disease [22,23,24]. In vivo models have demonstrated that the time interval between tumor excision and administration of chemotherapy is of great consequence, with chemotherapy given at the time of tumor removal having the greatest effect in controlling metastases [22]. Taken together, these studies provide a biological rationale for the administration of perioperative chemotherapy. This theory is further supported by clinical studies demonstrating the benefit of perioperative chemotherapy [25,26,27]. This benefit has been observed even with a single course of perioperative systemic therapy [27]. Targeting occult micrometastatic disease with anti-HER2 therapy as soon as possible after surgery can, therefore, enhance the effectiveness of systematic treatment.

These findings led to considerable investigation into establishing the significance of timely initiation of adjuvant therapy after breast cancer surgery. A large retrospective review by Lohrisch et al. found that delays of greater than 12 weeks in starting chemotherapy after surgery negatively impacted survival outcomes [6]. Another study, including 24,843 patients, had similar findings, concluding that delays of 91 days or more resulted in worse overall survival on pooled analysis. However, on subtype analysis, this effect was seen in triple-negative breast cancer (TNBC) but not in the HER2+ subtype [28]. However, the authors acknowledge that this lack of significance may be due to the relatively small number of HER2+ patients and the inconsistent use of trastuzumab-based therapy in this cohort [28]. Specifically, in HER2+ breast cancer, our findings are consistent with those of Gallagher et al., who reported that initiating adjuvant trastuzumab more than 6 months after breast cancer diagnosis was associated with significantly increased risks of relapse and mortality [13]. However, our study applied a shorter, clinically actionable threshold of 6 weeks.

These are indeed considerable delays, and investigating shorter time intervals consequently became the subject of great interest. Gagliato et al. [12], therefore, examined the impact of delaying adjuvant chemotherapy by ≥61 days, compared to those who commenced treatment in less than 30 days after surgery, finding a significantly worse OS in the former cohort. Of note, this finding was particularly significant in the trastuzumab-treated HER2+ subgroup (HR 3.09; 95% CI, 1.49 to 6.39, *p* = 0.002). However, there were no significant differences in survival observed in HER2+ patients who were treated with adjuvant regimens that did not include trastuzumab, nor were there differences between initiating trastuzumab treatment in the first 30 days compared to those who started treatment 31–60 days after surgery (HR 0.74; 95% CI, 0.35 to 1.54, *p* = 0.41). Other studies have also found that the consequences of delays are subtype-specific, with timely initiation of adjuvant therapy being of greatest importance in the HER2+ and TNBC subtypes [29,30].

In our study, we observed that even delays of greater than 42 days in starting adjuvant trastuzumab can negatively impact survival outcomes. While other studies have explored the effects of substantial delays, 6 weeks is a modest timeframe with real-world applications/relevance. These findings offer compelling evidence to support a range of clinical practice guidelines, which advise that adjuvant therapy for breast cancer should be started as soon as clinically feasible after definitive surgery [31,32], with the ESMO guidelines proposing this should ideally be done within 4–6 weeks [15]. A recent study found evidence to support this in the adjuvant treatment of TNBC [33]. Despite this, to our knowledge, this is the first study investigating the significance of initiating trastuzumab within a 6-week timeframe in HER2+ breast cancer.

Although clinical guidelines emphasize the importance of prompt initiation of adjuvant trastuzumab-based therapy, real-world implementation can be challenging. Factors such as delays in surgical recovery, coordination of multidisciplinary care, and limited oncology service capacity can all contribute to treatment delays [12,34]. Patient-related factors, including comorbidities, the need for psychological readiness, or logistical difficulties, such as travel, may also play a role [35,36]. These barriers underscore the need for streamlined care pathways and proactive scheduling to reduce avoidable delays and ensure timely therapy initiation.

This study is limited by its retrospective nature. While our results are compelling, with meaningful follow-up, we recognize that the reasons for delays in starting adjuvant therapy in clinical practice are often due to complications associated with surgery or patient comorbidities [37,38]. Unfortunately, our prospectively maintained database does not capture data relating to patient comorbidities and complications of surgery, with the possibility that these factors may be contributing to inferior survival outcomes. We also acknowledge that patient preferences and multidisciplinary team recommendations could not be accounted for. Additionally, a variety of adjuvant chemotherapy regimens used increased the heterogeneity of this cohort. Nonetheless, the results are striking, with significantly better OS, DFS, and DMFS associated with timely initiation of therapy. The strength of this study lies in its long follow-up time for a consistent cohort of HER2+ patients, with no significant histopathological or treatment differences between the groups.

In conclusion, this study demonstrated that delaying the initiation of adjuvant trastuzumab therapy in HER2+ breast cancer negatively impacts survival outcomes. This negative impact is observed even with modest delays of greater than 42 days, which are common in real-world clinical practice. Our results are striking, adding credence to current clinical practice guidelines, specifically in patients with HER2+ breast cancer. Clinicians should, therefore, strive to avoid postponing therapy whenever possible. These findings, along with similar results in prior studies, underscore the importance of avoiding delays in the administration of adjuvant trastuzumab whenever clinically feasible in order to optimize long-term patient outcomes.

## Figures and Tables

**Table 1:** Patient and clinical characteristics by TTAT.

			TTAT (Days)		
Characteristic		All (n = 227)	≤42 (n = 80)	>42 (n = 147)	*p*-Value *
Age at diagnosis	Mean ± SD (range); median	51.9 ± 13.4 (23–89); 48	52.1 ± 14.1 (31–81); 48.5	51.9 ± 13.1 (23–89); 48	0.9 ^†^
≤48 years	N (%)	114 (50.2%)	40 (50%)	74 (50.3%)	0.96 χ2
>48 years		113 (49.8%)	40 (50%)	73 (49.7%)	
Tumour size	Mean ± SD; median (mm)	31.5 ± 21.0; 26	28.5 ± 16.4; 26	33.2 ± 23.1; 27	0.11 ^†^
Pathological tumour stage	N (%)				0.6 χ2
T1		68 (30%)	23 (28.7%)	45 (30.6%)	
T2		123 (54.2%)	47 (58.8%)	76 (51.7%)	
T3		34 (15%)	9 (11.3%)	25 (17%)	
T4		2 (0.9%)	1 (1.3%)	1 (0.7%)	
Nodal status	N (%)				0.12 χ2
N0		104 (45.8%)	37 (46.3%)	67 (45.6%)	
N1		88 (38.8%)	25 (31.3%)	63 (42.9%)	
N2		16 (7%)	8 (10%)	8 (5.4%)	
N3		19 (8.4%)	10 (12.5%)	9 (6.1%)	
Hormone receptor status	N (%)				0.27 χ2
Positive		166 (73.1%)	55 (68.8%)	111 (75.5%)	
Negative		61 (26.9%)	25 (31.3%)	36 (24.5%)	
Tumour grade	N (%)				0.7 χ2
I		17 (7.5%)	7 (8.8%)	10 (6.8%)	
II		87 (38.3%)	28 (35%)	59 (40.1%)	
III		123 (54.2%)	45 (56.3%)	78 (53.1%)	
Lymphovascular invasion	N (%)				0.39 χ2
Negative		119 (52.4%)	35 (43.8%)	73 (49.7%)	
Positive		108 (47.6%)	45 (56.3%)	74 (50.3%)	
Type of surgery	N (%)				0.16 χ2
WLE		102 (44.9%)	41 (51.2%)	61 (41.5%)	
Mastectomy		125 (55.1%)	39 (48.8%)	86 (58.5%)	
Adjuvant regimen	N (%)				0.33 χ2
ACTH-like		119 (52.4%)	39 (48.8%)	80 (54.4%)	
TCH-like		14 (6.2%)	7 (8.8%)	7 (4.8%)	
Trastuzumab + Taxane	N (%)	62 (27.3%)	22 (27.5%)	40 (27.2%)	
Trastuzumab monotherapy		24 (10.6%)	11 (13.8%)	13 (8.8%)	
Other trastuzumab-based regimens		8 (3.5%)	1 (1.3%)	7 (4.8%)	
Radiation therapy	N (%)				0.5 χ2
Yes		170 (74.9%)	62 (77.5%)	108 (73.5%)	
No		57 (25.1%)	18 (22.5%)	39 (26.5%)	
Histological type	N (%)				0.4 χ2
IDC		208 (91.6%)	76 (95%)	132 (89.8%)	
ILC		10 (4.4%)	2 (2.5%)	8 (5.4%)	
Other		9 (4%)	2 (2.5%)	7 (4.8%)	

χ2 denotes Chi-square test; ^†^ denotes one-way analysis of variance (ANOVA) test; * denotes statistical significance. Abbreviations: TTAT, time to adjuvant trastuzumab; SD, standard deviation; WLE, wide local excision; ACTH, Doxorubicin, Cyclophosphamide, Paclitaxel, and Trastuzumab; TCH, Docetaxel, Carboplatin and Trastuzumab; IDC, invasive ductal carcinoma; ILC, invasive lobular carcinoma.

## Data Availability

Data used in this study can be made available on request to the corresponding author.

## Abbreviations

ASCO	American Society of Clinical Oncology
CAP	College of American Pathologists
CI	Confidence interval
DFS	Disease-free survival
DMFS	Distant metastasis-free survival
ESMO	European Society for Medical Oncology
HER2	Human epidermal growth factor receptor 2
HR	Hazard Ratio
OS	Overall survival
TNBC	Triple-negative breast cancer
TTAT	Time to adjuvant trastuzumab-based therapy
